# Enhanced bacterial cellulose production from spent coffee grounds: a dual-strategy synergy via mannose metabolism and pellicle inoculation

**DOI:** 10.1128/aem.02439-25

**Published:** 2026-05-05

**Authors:** Fan Yang, Ahmed K. Saleh, Lin Chen, Zhangjun Cao, Xingping Zhou, Feng F. Hong

**Affiliations:** 1 State Key Laboratory of Advanced Fiber Materials, College of Biological Science and Medical Engineering, Donghua University 528786 , Shanghai, China; 2 Scientific Research Base for Bacterial Nanofiber Manufacturing and Composite Technology, China Textile Engineering Society 571997 , Shanghai, China; Chalmers tekniska hogskola AB, Gothenburg, Sweden

**Keywords:** bacterial cellulose, *Komagataeibacter xylinus*, spent coffee grounds, mannose metabolism, biorefining, dual-strategy optimization

## Abstract

Mannose constitutes a principal component in numerous biomass hemicelluloses; however, its utilization by the cellulose-producing bacterium *Komagataeibacter xylinus* is inefficient. Consequently, during biorefinery and the transformation of biomass rich in mannose for the production of the high-value biological product, bacterial cellulose (BC), the biomass components cannot be fully utilized, leading to resource wastage. Moreover, inappropriate discharge of the fermentation effluent may also result in environmental pollution. This study addressed the underutilization of mannose in spent coffee grounds (SCGs) by introducing a fusion vector expressing mannokinase and phosphomannose isomerase into *K. xylinus* ATCC 23770, thereby enhancing mannose utilization in this strain model. SCG was pretreated with concentrated sulfuric acid to disrupt crystalline structures, followed by enzymatic hydrolysis to extract saccharides. When culturing in SCG hydrolysate, BC pellicle-derived inoculum achieved 1.2-fold higher BC yield than broth inoculum, attributed to BC matrix protection. The engineered strain *K. xylinus* CGMCC 31806 demonstrated superior mannose assimilation, yielding 1.5-fold more BC than wild type in simulated SCG medium. In authentic SCG medium without extra nitrogen addition, BC production reached 3.80 g/L, a 1.2- to 2.5-fold increase. The obtained BC exhibited enhanced mechanical properties with 40–84% higher tensile strength and 20–75% increased Young’s modulus. This study validates a dual strategy combining agro-waste valorization and targeted strain modification, establishing a collaborative optimization framework linking biomass feedstocks, microbial engineering, and bioprocessing for cost-effective BC production. Future research would utilize existing commercial high-yield strains to replicate the expression strategy of this fusion protein, in order to achieve truly efficient industrial production.

**IMPORTANCE:**

The inefficient utilization of mannose, a major hemicellulose component prevalent in biomass by *Komagataeibacter xylinus* represents a significant bottleneck in the sustainable, cost-effective bioproduction of bacterial cellulose (BC). This underutilization would lead to wasted resources and potential environmental pollution from fermentation effluents. The study directly addresses this critical challenge by pioneering a synergistic “Feedstock-Strain-Process” integration strategy in advancing BC industrialization. We demonstrate the significant impact of engineering *K. xylinus* for enhanced mannose assimilation coupled with optimized biorefinery of agro-industrial waste, taking mannose-rich spent coffee grounds, as the representative raw material. This integrated approach not only valorizes abundant agricultural waste streams significantly boosting BC yields under nutrient-limited conditions but also generates BC with superior mechanical properties. The study establishes an actionable blueprint for advancing BC industrialization through collaborative optimization of biomass feedstocks, targeted microbial engineering, and process innovation, aligning with the goals of the circular bioeconomy and sustainable material production.

## INTRODUCTION

Bacterial cellulose (BC), a natural polymer like plant cellulose, consists of d-glucose units connected by β−1,4 glucosidic bonds (1). It is used in textiles, food, medical products, and functional materials. Currently, there is no specific bacterial strain designated for BC production in a commercial context. Instead, different manufacturers have developed their own patented bacterial strains, among which bacteria of the genus Komagataeibacter are widely applied. *Komagataeibacter xylinus*, mainly metabolizing glucose and fructose, is widely used for commercial BC production (2). However, high production costs restrict its widespread application. Conventionally, either genetic modification of BC-producing strains to enhance yield, or development of low-cost culture media has been applied (3). In BC’s industrial production, the culture medium accounts for about 30% of the total cost (3). The affordable media often derive from industrial and agricultural residues, with particular interest in cellulosic and lignocellulosic wastes, such as hydrolysates from spruce fragments (4), sweet sorghum bagasse (5), and cotton textile waste (6). Table S1 lists relevant literature and BC productivity.

Genetic modification of *K. xylinus* often aims to increase BC production, such as the heterologous expression of the *vgb* gene increasing production under hypoxic stress (7). Some studies have also focused on the carbon source utilization ability of *K. xylinus*. For example, overexpressing the sucrose phosphorylase gene from *Leuconostoc mesenteroides* in an *Acetobacter* strain increased BC production from sucrose by 27% (8), and expressing a mung-bean sucrose synthase in *K. xylinus* doubled BC production (9). However, these BC-yield validations were in laboratory semi-synthetic media and not applied to biomass media. Also, integrating the *Escherichia coli* β-galactosidase (*lac*Z) gene into *K. xylinus* ITDI enabled it to convert lactose to cellulose, with BC concentrations ranging from 0.78 to 1.82 g·L^−1^ (10). Whey, a former cheese byproduct, is now valuable in the food industry. Lactose is primarily found in dairy products and is scarce in other biomass waste. So, there is a gap between these studies and practical production.

Coffee is one of the most consumed beverages globally, with an annual consumption of 166,346,000 bags (about 10 million tons) (11). For each ton of coffee beans processed, about 650 kg of spent coffee grounds (SCGs) are produced (12). Historically, SCG was used as boiler fuel or for fertilizer/landfilling (13), but this causes environmental issues. In recent years, SCG has been developed and applied in producing bioethanol, biopolymers, bioadsorbents, and biocomposites (13, 14). SCG mainly consists of lignocellulose, proteins, and lipids, with the most abundant polysaccharide being galactomannan (15). Table S2 summarizes current hydrolysis methods and sugar recovery rates from SCG. SCG has been used in BC production, usually through soaking solutions. For example, Agüero et al. produced BC from coffee grounds soaking solution (16), and Candra et al. used SCG as a nitrogen source and produced BC as a byproduct (17). Caffeine in SCG should enhance BC production by *K. xylinus* as it inhibits the activity of phosphodiesterase, increasing cyclic guanylate and boosting BC production (18). So far, there are no studies on using SCG hydrolysate in BC production.

Although metabolic analysis and biomass cultivation techniques of *K. xylinus* have made significant progress over the past 5 years (19), these achievements are not directly related to the present study. To date, there has been insufficient focus on the carbon source spectrum research of this bacterium, and coffee residue hydrolysate has never been used for BC production. Research on improving carbon source utilization of this strain through synthetic biology techniques has only been reported in 1998 (8), 1999 (9), and 2004 (10), and the number of research reports is very scarce. Mannose, an important hemicellulose component, has greater potential as a fermentation carbon source for large-scale BC production (4, 20). However, the low efficiency of *K. xylinus* in utilizing mannose can lead to resource waste and environmental pollution when biomass is used as an inexpensive culture medium (2, 19). In *K. xylinus* using mannose, there are two limiting steps: mannose phosphorylation and the isomerization of phosphomannose (21). It has been shown that glucose, mannose, and glucosamine act as substrates for the *K. xylinus* glucose kinase*,* although the catalytic performance is suboptimal (22). Griffin et al. found that the *ace*F gene in *K. xylinus* encodes phosphomannose isomerase (PMI) and GDP-mannose pyrophosphorylase; however, overexpression of *ace*F did not significantly enhance the catalytic ability of PMI (23).

Mannose kinase (MAK) and phosphomannose isomerase (PMI) from *E. coli* K-12 demonstrate high catalytic activity toward substrates. MAK and PMI were introduced into *Ralstonia eutropha* in the form of fusion proteins to broaden the substrate range for polyhydroxyalkanoate production (24). The genes encoding MAK and PMI were individually linked to the Pnk promoter for transcriptional expression (non-fusion protein) to promote BC synthesis using mannose as a substrate, resulting in an 84% increase in BC yield (25). However, compared to dual-promoter expression plasmids, fusion protein expression plasmids should have a simpler structure (26), eliminating the need to design the relative positions and strengths of two promoters and thereby achieving more stable gene expression. Additionally, fusion expression plasmids are more space-efficient and would impose smaller burdens on the host (27). Based on the premise that the folding of the two proteins does not cause mutual interference, the use of fusion proteins could enhance the metabolic efficiency of PMI and MAK, increase the likelihood of interaction between phosphorylated mannose and the MAK active site, and further enhance mannose metabolism. To make *K. xylinus* better adapt to the mannose-rich SCG medium, MAK and PMI were therefore introduced as a fusion protein to alleviate mannose metabolic limitation in the study.

This study demonstrates innovation in the field of high-value utilization of biomass, with its core contribution lying in the first proposal and implementation of the concept of “strain-medium adaptation.” Specifically, using SCG rich in mannose as a model substrate, combined with targeted metabolic enhancement strategies for engineered strains, this study first strengthened the mannose metabolic pathway of *K. xylinus*, significantly improving the utilization efficiency of complex biomass. The complete chain from gene modification to practical application verification not only fills the research gap in the field of efficient mannose metabolism of *K. xylinus*, but also would provide an economically feasible approach for developing culture media using abundant and low-cost biomass resources. Furthermore, this study conducted an in-depth evaluation of the impact of different inoculation methods on the adaptability of *K. xylinus* on the SCG hydrolysate medium. BC pellicle and broth were separately inoculated into the hydrolysate media, the fermentation process was monitored, and the physicochemical properties of the product BC were analyzed. The “strain-medium adaptation strategy” integrates multiple key steps, including waste resource conversion and high-value utilization, microbial genetic modification (strengthening mannose metabolism in *K. xylinus*), and optimization of fermentation production processes (comparison of inoculation methods). This strategy not only provides a new technological paradigm for the efficient utilization of biomass waste but also would promote the industrialized production of BC, thereby contributing to the achievement of the “dual carbon goals.”

## MATERIALS AND METHODS

### Optimization of engineered bacteria to enhance the mannose metabolic pathway

Phosphorylation and isomerization restrict the utilization of mannose by *K. xylinus*. To eliminate this constraint, a new fusion-protein expression plasmid, pBBR1MCS2::*pmi-mak*, was constructed. In accordance with the report by Sichwart et al. (24), the linker peptide was synthesized. Subsequently, the plasmid was introduced into *K. xylinus* 23770 via electroporation. To individually assess the contributions of *pmi* and *mak* to mannose utilization, control plasmids pBBR1MCS:*pmi* and pBBR1MCS:*ma*k were constructed. All the bacterial strains and plasmids employed in the current study are presented in Table S3. The new engineered bacterium was named *K. xylinus* CGMCC 31806 (now stored in China General Microbiological Culture Collection Center).

### *K. xylinus* CGMCC 31806 expression verification and BC production

Insertion fragment in the plasmid pBBR1MCS2::*pmi-mak* was sequenced using universal M13/PUC-forward and reverse primers. To confirm the recombinant plasmid’s integrity, a plasmid extraction kit was used to extract the plasmid of the engineered strain. Digestion was validated with the restriction enzymes *BamH*I and *Xho*I. Centrifuge to collect bacterial cells, then lyse the cells and centrifuge again to collect the supernatant. The 3′ end of the inserted fragment is labeled with polyhistidine, and the gene expression product is purified by Ni-NTA His binding resin (7sea Biotech, Shanghai, China). Protein samples were separated in 12.5% (wt/vol) SDS-polyacrylamide gel (28) and stained with Coomassie Brilliant Blue R-250.

To compare the effect of plasmid optimization on BC production, *K. xylinus* ATCC 23770 and CGMCC 31806 were cultured in mannose-based H&S medium for 10 days. BC was harvested and purified daily, dried at 105°C for 30 min, and weighed. This process was repeated until the weight difference between measurements was less than 0.15%, indicating reliability. Samples were cooled to room temperature before weighing. All experiments were performed in triplicate.

### Concentrated sulfuric acid pretreatment of SCG

Studies have shown that despite variations in the individual components of different SCG varieties or treated SCGs, the component types are similar (29). For instance, Nguyen et al. hydrolyzed SCG from Starbucks coffee in Gwangju to yield monosaccharides, but did not analyze the influence of the origin and processing of the beans (30). Other studies also analyzed SCG from coffee shops or vending machines without assessing either the sources or processing procedures of the beans (31–33). The present investigation did not focus on the caffeine, chlorogenic acid, or other low molecular-weight components of SCG, but only examined whether the SCG hydrolysate can serve as a medium for culturing the mannose metabolism-enhanced bacterial strain. The SCG used in the study was collected from a Starbucks coffee shop in Shanghai and was used to culture the engineered bacterium. The used coffee waste was dried at 80°C for 48 h and stored under seal at room temperature in a cool, dry place for further experiments. All reagents for subsequent treatment were derived from Sinopharm Chemical Reagent Co. (Shanghai, China). Employing the Van Soest method (34), it was determined that the untreated SCG contained 26.9% cellulose, 42.1% hemicellulose, and 6.2% lignin. This result is in accordance with the previously reported cellulose (12.6–47.3%) and hemicellulose (32.0–53.0%) content in SCG (35).

Dried SCG was soaked in sulfuric acid at concentrations of 50%, 60% and 70% to achieve a 1:1 (wt/vol) solid-liquid ratio, and treated at 30°C for 1 h. Post-pretreatment, the pH was adjusted to neutral with sodium hydroxide, followed by filtration and drying of the residue. The treated solids were analyzed by X-ray diffractometer (XRD, D/max-2550VB+/PC, Rigaku, Tokyo, Japan).

No detoxification was performed after pretreatment with concentrated sulfuric acid to avoid removing microbial growth inhibitors from the hydrolyzates.

### Enzymatic hydrolysis and optimization of SCG

Hemicellulase and cellulase (Shanghai Yuanye Bio-Technology Co., Ltd, Shanghai, China) were used together to saccharify the residue remaining after pretreatment of SCG. To determine the optimal pH and temperature for their interaction, enzyme activity was assessed across pH 2.5–6.0 and temperatures 20–80°C. The solid-liquid ratio and enzyme dosage were optimized based on the final recovered sugar yield. The yield of recovered sugars during the optimization process of enzymatic hydrolysis was determined using the anthrone-sulfuric acid colorimetric method to calculate the sugar concentration in the medium after filtration through a 0.22 μm membrane filter (36).

Calcium hydroxide adjusted the pH of the filtrate and stirred, removing the precipitate. The filtrate was remixed with the undissolved residue after pretreatment, and the concentrations of furfural and polyphenol were determined. Enzymatic hydrolysis was carried out under suitable conditions. The pretreatment and enzymatic hydrolysis processes for SCG are illustrated in Fig. S1. Monosaccharide analysis of the hydrolysate was performed on a Thermo ICS5000 ion chromatography system (ICS5000, Thermo Fisher Scientific, USA) equipped with a Dionex CarboPac PA20 column (150 × 3.0 mm, 10 μm). Monosaccharides were identified by comparing their retention times with those of standard monosaccharides. The total sugar concentration of the hydrolysate was determined by using the 3,5-dinitrosalicylic acid (DNS) colorimetric method.

The recovered sugar yield (*Ys*, %) was calculated using the following equation. *C* is the concentration of the component in the liquid phase (g/L), *M* is the theoretical total sugar mass of SCG dry matter (g), and *V* is the volume of liquid solution employed (L).


$$Ys=\left(C\times \frac{V}{M}\right)\times 100\%$$

The theoretical total sugar mass of SCG dry matter (*M*) includes:


$$M=k\cdot M_{SCG}\left(C_{C}+C_{H}\right)+M_{P}$$

*M*_SCG_ is the mass of dry matter in SCG. *C*_*C*_ is the cellulose content (%) in SCG, and *C*_*H*_ is the hemicellulose content (%) in SCG. Cellulose or hemicellulose is composed of polysaccharide chains formed by dehydration of monosaccharides through glycosidic bonds; the molar mass of each repeating unit is 162 g/mol. In SCG, cellulose or hemicellulose is primarily composed of glucose, mannose, and galactose. These three monosaccharides are isomers (C_6_H_12_O_6_) with a molar mass of 180 g/mol each. Therefore, the coefficient *k* for hydrolysis of the polysaccharide chain into monosaccharides is 1.111. *M*_*P*_ is a polysaccharide extracted by using neutral detergent, with any remaining pectin or oligosaccharides in the SCG being completely washed out (34). The phenol acid anthrone colorimetric method was used for determining the content of the dissolved polysaccharide.

The concentrations of furfural and total phenols in the hydrolysate were determined by spectrophotometry. The reaction of aniline with furfural in concentrated acetic acid yields a red compound, C_4_H_3_OCH(C_6_H_5_NH)_2_, featuring a distinct absorption peak at 520 nm. This peak remains unaffected by other aldehydes, using furfural as the standard for curve plotting (37). In alkaline conditions, polyphenols react with phosphotungstate and molybdic acid in the Folin-Ciocalteu reagent, producing a blue solution. The absorbance of this solution at 760 nm is directly proportional to the total phenol content, with gallic acid used as the standard for calibration (38). SCG was soaked in deionized water overnight at the same solid-liquid ratio as the hydrolysate, then filtered to prepare the SCG extract, which was used to measure inherent inhibitors.

### Production of BC in SCG hydrolysate medium

Due to the nutrient-rich composition of SCG, no additional nitrogen source was added to the hydrolysate medium, and the initial pH was adjusted to 5.2 using sulfuric acid or sodium hydroxide. In order to observe the impact of complex monosaccharides in SCG on microbial growth, an SCG simulated medium (referred to as the simulated medium) was set up as well. Prepare a simulated hydrolysate with the same sugar components based on the results of the monosaccharide analysis of the SCG hydrolysate, while other components are the same as those in the H&S medium. Wild-type (WT) *K. xylinus* ATCC 23770 (WT) and engineered *K. xylinus* CGMCC 31806 (EB) were inoculated into SCG hydrolysate in the form of culture broth and BC pellicle, respectively. However, bacterial seeds were inoculated into simulated medium only in the form of culture broth. To compare BC yield between broth and pellicle inoculation, inoculation amounts must be consistent.

#### BC pellicle inoculation of SCG hydrolysate [SCG(P)]

Activated bacterial strains were inoculated into shake flasks and incubated overnight to obtain primary seed broth, then inoculated into a 24-well plate at 5% (vol/vol), and statically cultured overnight to form BC pellicle. To calculate the number of cells in the BC pellicle, a piece of BC pellicle was dispersed using a household fruit and vegetable grinder to create a crude fiber suspension. This suspension was diluted to 15 mL and processed with a high-pressure homogenizer (GA-10H, LHF Nano Technology, China) at 1,800 bar, cycled three times, yielding a mixture of BC homogenate and bacterial cells. Cell numbers in the BC homogenate were determined via fluorescent staining (39). In the case of BC pellicle inoculation, a section of BC pellicle was employed to inoculate the SCG hydrolysate. The BC generated through the pellicle inoculation method is designated as SCG(P).

#### Liquid inoculation of SCG hydrolysate [SCG(L)]

The primary seed broth was inoculated into a shake flask at the same inoculum level and incubated overnight to obtain the secondary seed broth. For liquid inoculation, cells from secondary seed broth were centrifuged, diluted to match BC pellicle inoculation cell counts, and then inoculated into SCG hydrolysate. In the case of liquid inoculation, cells from the secondary seed broth were subjected to centrifugation, diluted to align with the cell counts required for BC pellicle inoculation, and subsequently inoculated into the SCG hydrolysate. The BC produced via the liquid inoculation method is designated as SCG(L).

The resultant BC was treated with 1% (wt/vol) aqueous NaOH solution at 80°C for 2 days, washed with deionized water until the pH of the water was neutral, and then dried at 105°C to constant weight. All counting operations were repeated three times. Changes in pH, residual sugar, free cell count, cell count in BC, and BC yield during fermentation were observed and recorded. Post-harvest, BC pellicle was analyzed for surface morphology, ATR-FTIR, XRD, and mechanical strength, as previously reported (2).

### Statistical analysis

Statistical significance was assessed using *t*-tests. Results demonstrating *P* values < 0.05 were annotated with (*) to denote significance. Data points achieving stricter thresholds of *P* < 0.01 and *P* < 0.001 received (**) and (***) markings, respectively. Conversely, (#) symbols designated findings where *P* values exceeded 0.05, indicating non-significant outcomes.

## RESULTS AND DISCUSSION

### Characterization of *K. xylinus* CGMCC 31806

**Fig 1:**
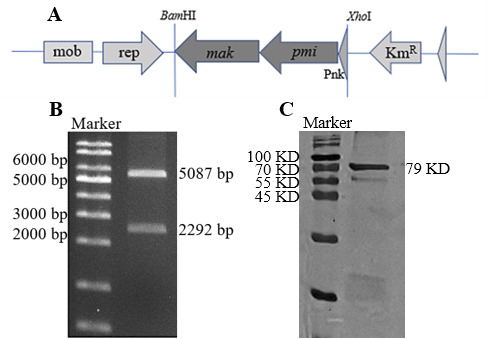
(**A**) Physical map of the constructed plasmid pBBR1MCS2::*pmi-mak*. Relevant cleavage sites and structural genes are indicated. KmR, kanamycin resistance cassette; mob, mobilization site; rep, origin of replication; *mak*, gene encoding a mannose kinase (MAK) from *E. coli* (accession no. NP_414928.2); *pmi*, gene encoding a phosphomannose isomerase (PMI) from *E. coli* (accession no. NP_416130.3). (**B**) The transformed plasmid was extracted for enzymatic digestion and analysis. (**C**) SDS-PAGE analysis of the purified protein of recombinant *K. xylinus* CGMCC 31806. DNA and protein markers used for size comparison.

Figure 1A demonstrates that in this study, the *pmi-mak* gene was inserted between the *Xho*I and *BamH*I sites of the digested plasmid pBBR1MCS-2, aligned with the Pnk promoter. The resultant recombinant plasmid was named pBBR1MCS::*pmi-mak*. Since the Pnk promoter is constitutive, both genes were intended to be expressed constitutively. The plasmid was introduced into *K. xylinus* ATCC 23770 through electroporation, and the kanamycin-resistant transformant *K. xylinus* CGMCC 31806 was subsequently isolated after screening.

Figure 1B and C show the DNA transformation analysis and the SDS-PAGE analysis of the enzymes. The plasmid of the engineered bacterium *K. xylinus* EB was extracted using a kit to obtain the 7,379 bp plasmid. The *BamH*I and *Xho*I digestion was verified, which, as expected, showed two DNA fragments, the 2,292 and 5,087 bp fragments (Fig. 1B). A 79 kD band of the fusion enzyme after purification on the Ni-NTA His Bind Resin can be clearly seen in the SDS-PAGE electrophoretic map (Fig. 1C).

The wild-type strain WT and engineered bacteria EB (harboring pBBR1MCS::pmi-*mak*), EB-*pmi* (harboring pBBR1MCS:*pmi*), and EB-*mak* (harboring pBBR1MCS:*mak*) were statically incubated in mannose-based H&S media for a duration of 10 days. Ultimately, the yield of EB (0.42 g/L) was 2.1-fold higher than that of WT (0.20 g/L). Nevertheless, the BC yield of the control groups EB-*mak* (0.24 g/L) and EB-*pmi* (0.22 g/L) (Fig. 3C) did not exhibit significant disparities when compared to WT. This suggests that both phosphorylation and isomerization serve as rate-limiting steps in mannose metabolism; interaction with a single rate-limiting step fails to enhance the mannose utilization capacity. Conversely, PMI and MAK, functioning as fusion proteins, possess a potent catalytic ability to direct a greater amount of mannose into the metabolic cycle. Compared to the dual-promoter expression plasmid, the fusion protein expression plasmid used in this study is simpler to construct and eliminates the need to design the relative position and strength of the two promoters, leading to more stable gene expression. Additionally, the fusion expression plasmid is more space-efficient and imposes a lesser burden on the receptor (40). Under the premise that the folding of the two proteins does not interfere with each other, the fusion protein can enhance the metabolic efficiency of PMI and MAK, increasing the likelihood of phosphorylated mannose interacting with the active site of MAK and further promoting mannose metabolism.

### Concentrated sulfuric acid pretreatment of SCG

Figure S2 presents an XRD analysis demonstrating the effects of treatment with concentrated sulfuric acid at varying concentrations. Higher concentrations of sulfuric acid resulted in lower peak values in the samples when compared to untreated SCG, suggesting an increase in the amorphous regions and a reduction in crystallinity, aligning with anticipated outcomes. SCG treated with 70% sulfuric acid has the lowest crystallinity (9.07%). This increase in amorphous structure facilitates easier contact with the enzyme active site, leading to more straightforward hydrolysis.

The complex structure of SCG complicates the extraction of sugars from its polysaccharides, making the choice of an appropriate pretreatment method crucial. For instance, Choi et al. used a popping pretreatment at 1.47 MPa, while Ravindran et al. employed a potassium permanganate-assisted ultrasonic pretreatment for coffee grounds (13). These methods require sophisticated equipment and environments, leading to relatively high processing costs. In this study, SCG was pretreated with concentrated sulfuric acid, known for its cellulose-swelling effects. Chen et al. reported that concentrated sulfuric acid is highly effective at dissolving cellulose, with the solubility of cotton cellulose reaching up to 300 g/L. The dissolution process involves the disruption of hydrogen bonds within the cellulose molecules by H^+^ cations, leading to the breakdown of the crystalline structure, a reduction in the degree of polymerization, and an increase in the compliance of the molecular chains (41). Additionally, the reaction of concentrated sulfuric acid with the aromatic rings in lignin not only disrupts the complex structure of lignocellulose but also diminishes the lignin’s physical barrier effects on subsequent hydrolysis processes (42).

This study used 70% (wt/vol) sulfuric acid to pretreat SCG with a solid-to-liquid ratio of 1:1. For each gram of SCG processed, 0.7 g of 100% H_2_SO_4_ is required. Based on the yield of reducing sugars (Table 1), it is calculated that 0.875 g of 100% H_2_SO_4_ is needed for every 1 g of reducing sugar produced. Wang et al. used 5.5% H_2_SO_4_ (wt/vol) pretreatment at 100°C with a solid-to-liquid ratio of 1:5, achieving an 81.5% sugar recovery rate and a reducing sugar yield of 563 mg/g (43). This means that 0.3 g of 100% H_2_SO_4_ is consumed per gram of SCG processed, and 0.533 g of 100% H_2_SO_4_ is needed for every 1 g of reducing sugar produced. Although the acid consumption for pretreatment with 70% (wt/vol) sulfuric acid is slightly higher, the concentrated sulfuric acid pretreatment requires lower reaction conditions and does not require detoxification. Concentrated sulfuric acid pretreatment just requires milder reaction conditions (physiological temperature 30°C for 1 h), unlike the pretreatment of dilute sulfuric acid under high temperature and pressure. Pretreatment of lignocellulose with concentrated sulfuric acid is a common treatment method. Uzyol and Sacan used 72% (wt/vol) sulfuric acid to pretreat algal starch for the production of BC without detoxification, achieving a yield of 0.157 g⋅L^−1^⋅d^−1^ (44). Kovalcik et al. used 4% (wt/vol) dilute acid hydrolysis of SCG (100°C for 2 h) to produce polyhydroxyalkanoates, and found that without the detoxification treatment of an adsorbent, the hydrolysate completely inhibited the growth of the bacterium *Halomonas halophila* (45). Many studies have shown that dilute sulfuric acid hydrolysis requires detoxification steps to ensure normal subsequent fermentation (46). Therefore, although concentrated sulfuric acid pretreatment requires more acid, less energy consumption and relatively simple treatment processes are applied.

**TABLE 1:** Monosaccharide analysis of the enzymatic hydrolysate of SCG^*a*^

Monosaccharide type	Sugar concentration (g/L)	Percentage (%) in total sugar
Glucose	4.12 ± 1.05	20.4
Mannose	11.23 ± 2.24	56.1
Arabinose	0.57 ± 0.04	2.8
Galactose	4.08 ± 0.76	20.7
Xylose	–	–
Fructose	–	–
Ribose	–	–
Fucose	–	–
Rhamnose	–	–
Total sugar	20.0 ± 1.81	100%
*Ys* of hydrolysis	84.7%	

^*a*^ –, the component is absent or below a detectable concentration. The solid-liquid ratio is 1:40. *Ys* (%), recovered sugar yield. (mentioned in “Enzymatic hydrolysis and optimization of SCG”). Percent (%) = concentration of each monosaccharide (g/L)/concentration of total sugar (g/L).

### Enzymatic hydrolysis of SCG and optimization of conditions

**Fig 2:**
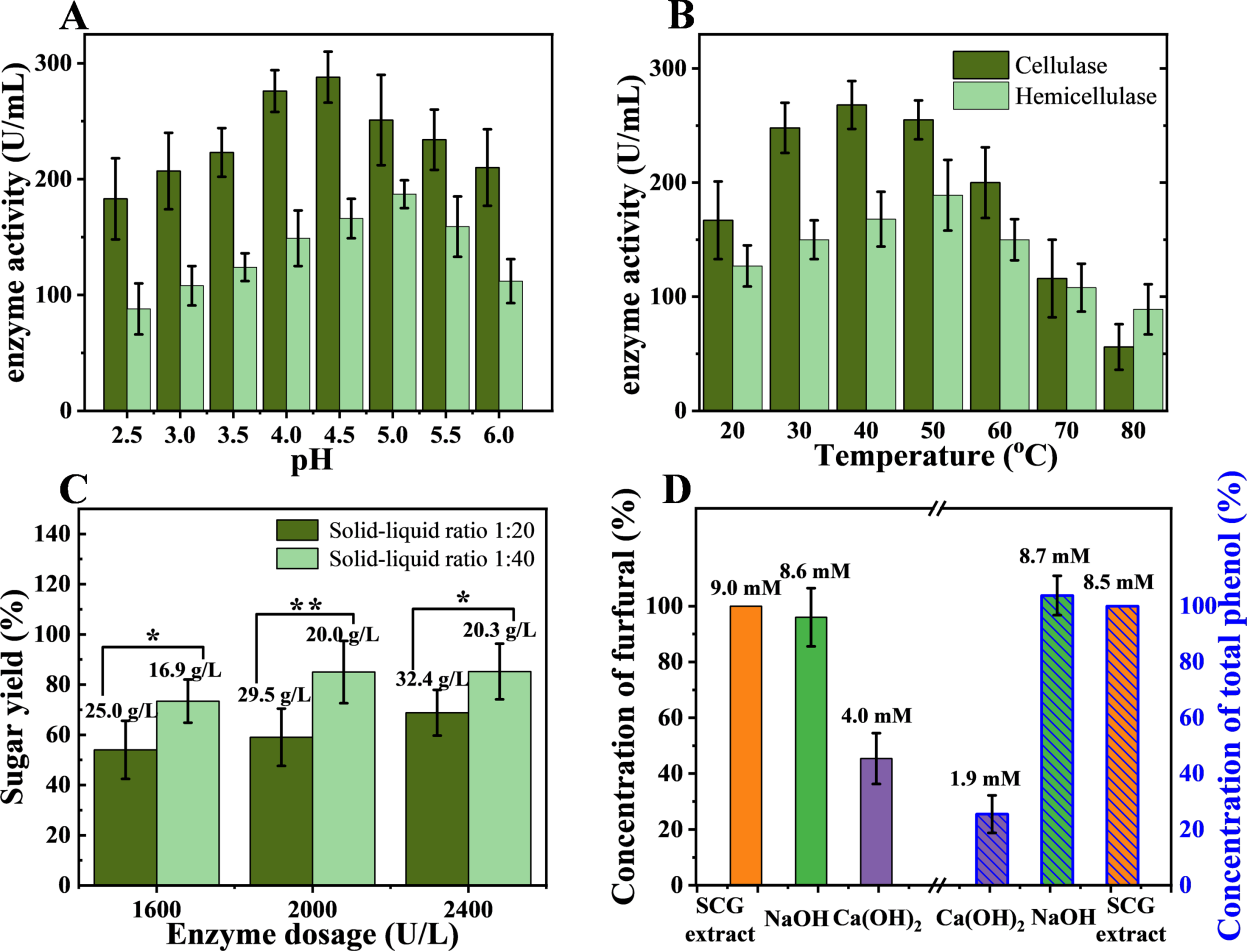
(**A**) Optimum pH for catalysis of cellulase and hemicellulase. (**B**) Optimum temperature for catalysis of cellulase and hemicellulase. (**C**) Relationship between enzyme addition, solid-liquid ratio, and sugar yield. (The figures above the columns represent the sugar concentration of the hydrolysate.) (**D**) Relative and absolute concentrations of furfural and total phenol in SCG extract and those after neutralization of sodium hydroxide/calcium hydroxide. *P < 0.05; **P < 0.01.

As depicted in Fig. 2A and B, the optimal pH and temperature for cellulase and hemicellulase are closely aligned. Cellulase performs best at a pH of 4.0–5.0 and a temperature range of 30–50°C, whereas hemicellulase has an optimal pH of 4.5–5.5 and thrives at temperatures between 40°C and 60°C. Based on these findings, a reaction pH of 5.0 and a temperature of 50°C were selected for the experiments. Figure 2C illustrates the relationship between the amount of enzyme added, the solid-liquid ratio, and the sugar yield. At enzyme addition levels of 2,000 and 2,400 U/mL, the sugar yields are comparable. Although a higher sugar yield was observed when the solid-to-liquid ratio was 1:20, the sugar conversion rate was lower. For instance, when the enzyme addition amount was 2,000 U/mL, the hydrolyzed mixture with a solid-to-liquid ratio of 1:20 had a sugar concentration of 29.5 g/L, yet the sugar recovery rate was merely 59%. In comparison, the sugar recovery rate for a solid-to-liquid ratio of 1:40 reached 84.7%. Therefore, the selection of a higher sugar recovery rate is crucial for maximizing raw material conservation and improving cost-effectiveness. Consequently, the selected enzyme addition level is 2,000 U/mL, and the solid-liquid ratio is 1:40. The monosaccharide composition of the SCG hydrolysate is shown in Table 1. Based on the calculations, the total sugar concentration in the hydrolysate was 20.0 g/L, with the sugar recovery rate (*Ys*) of 84.7%. Compared to other methods for the hydrolysis of SCG (Table S2), despite the relatively lower hydrolysis efficiency obtained in this study, the conditions used for the concentrated sulfuric acid pretreatment are mild and do not require the use of high temperature and pressure. SCG serves as a model for validating engineered strains, highlighting the importance of enhancing microbial metabolism of the carbon source for resource utilization and environmental protection. While the utilization of agricultural and industrial waste as culture media recycles resources, the organic matter in the waste liquid after fermentation may still pollute the environment. This study used mannose, commonly found in hemicellulose, as an example. Enhancing the capacity for mannose metabolism in engineered strains enables greater utilization of biomass resources by bacteria, thereby saving raw material costs, reducing the discharge of fermentation waste liquid, and potentially saving costs in the treatment of the waste liquid. This strategy can not only enhance the efficiency of resource conversion but may also benefit the environment.

Figure 2D details the changes in furfural and polyphenol concentrations after adjusting the pH of the pretreated mixture using sodium hydroxide and calcium hydroxide. Sodium hydroxide has minimal impact on furfural and polyphenol levels, while calcium hydroxide effectively reduces their concentrations after pH adjustment. Many agricultural waste materials inherently contain compounds like polyphenols, and the hydrolysis process may also generate furfural and other toxic compounds. Zou et al. reported that BC production with *K. xylinus* ATCC 23770 was reduced by 40–54% due to the presence of 10 mM furfural and by about 35% under the influence of 2 mM phenol (coniferaldehyde or vanillin) (47). As shown in Fig. 2D, the hydrolysate contained approximately 4.0 mM of furfural and about 1.9 mM of total phenols. Furfural’s inhibitory effect primarily disrupts cell membranes, inhibits the electron transport chain, and causes DNA damage (47). At 4.0 mM, furfural may delay the logarithmic phase and reduce BC synthesis efficiency by requiring more time for cells to produce furfural dehydrogenase and other detoxifying enzymes. Polyphenols, at 1.9 mM, alter membrane permeability and interfere with signaling pathways, forcing cells to allocate carbon sources to antioxidant synthesis, which limits growth resources (47). The impact of different phenolic compounds on bacterial growth varies. When the concentration of coniferaldehyde exceeds 1.5 mM, it significantly affects the rate of glucose consumption. Ferulic acid at 2 mM does not have a noticeable negative impact on BC growth. When the concentration of vanillin exceeds 0.5 mM, it significantly affects cell viability (48). Other prevalent inhibitors, such as fatty acids, exert a negligible influence on *K. xylinus. K. xylinus*, an acetic acid bacterium, can tolerate lower pH levels and metabolize organic acids. Zhang et al. assessed the impact of acetic acid and levulinic acid at concentrations spanning from 0 to 250 mM on the growth of *K. xylinus*. Intriguingly, up to 175 mM acetic acid (inclusive of 175 mM), there was no discernible effect on the glucose consumption rate of the bacteria (3.1 g/(L·d)). However, at concentrations exceeding 175 mM, the glucose consumption rate marginally declined to 2.6 g/(L·d). The alterations in BC yield exhibited a similar pattern, with yields approximately 3.5 g/L at or below 175 mM and 3.1 g/L above 175 mM. The effect of levulinic acid was also inconsequential. At a concentration of 250 mM, the glucose consumption rate was identical to that of the control group without the addition of levulinic acid (3.1 g/(L·d)), and the BC yield was also comparable (3.5 g/L) (49). Consequently, in this study, only furfurals, which are readily generated during acid hydrolysis, and phenolic compounds, which significantly affect the growth of *K. xylinus*, were emphasized. Although neutralization and filtration with Ca(OH)_2_ can effectively reduce inhibitors, it also leads to the loss of potential resources. Future research will explore biological approaches, such as oxidase catalysis, to transform inhibitors like furan aldehydes into harmless and potentially useful intermediates, thereby enhancing resource value.

### Inoculation of hydrolysate and fermentation monitoring of production BC

**Fig 3:**
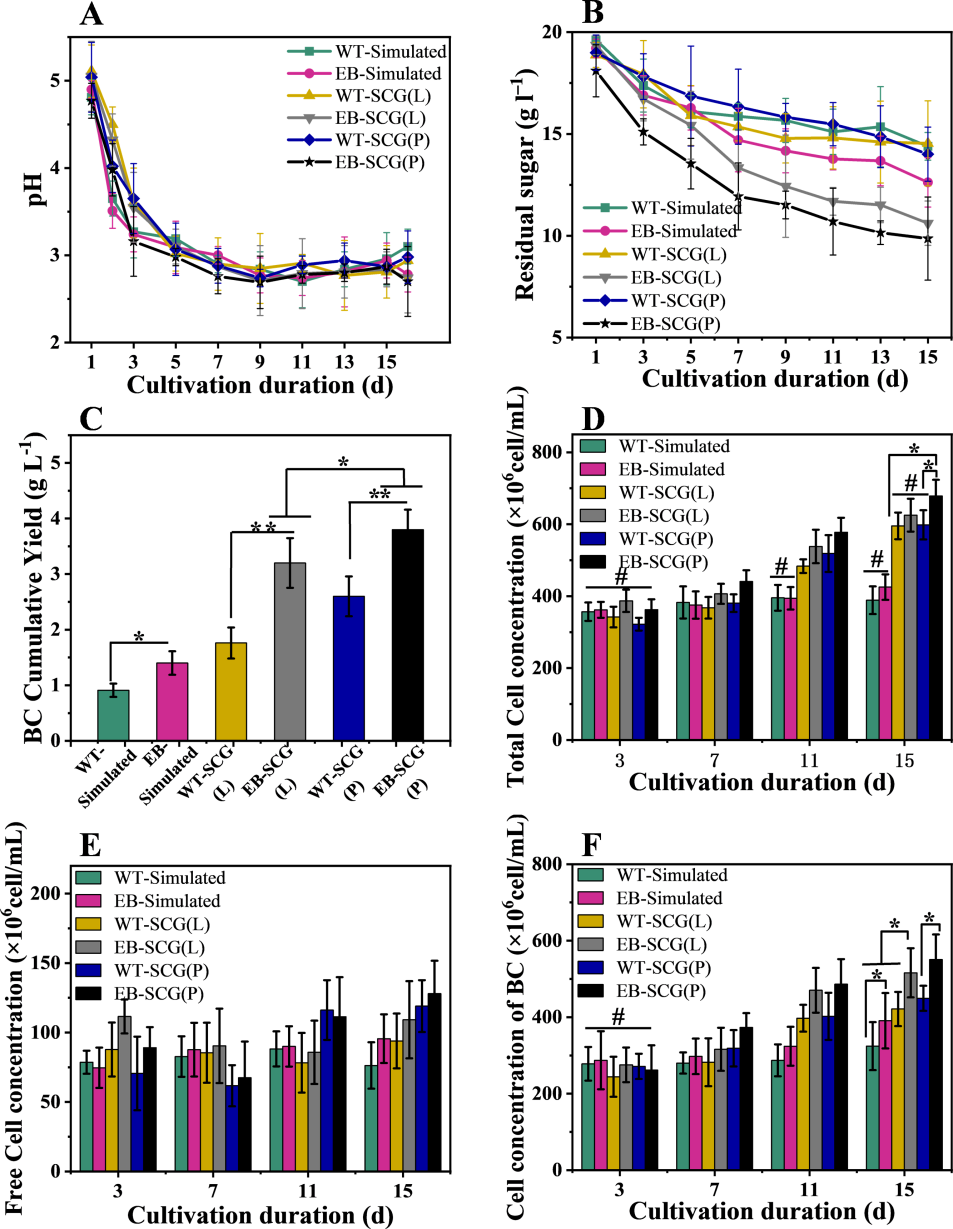
Time course of cultivation of wild-type strain and engineered strain in simulated and SCG hydrolysate media using the inoculation in the form of liquid and BC pellicle: (**A**) pH, (**B**) residual sugar, (**C**) BC cumulative yield, and (**D–F**) cell number changes. L, liquid inoculation. P, BC pellicle inoculation. Simulated, simulated medium. *P < 0.05; **P < 0.01; *#P* > 0.05.

The strains WT and EB were cultured in simulated medium and SCG hydrolysate medium (referred to as SCG medium) for 15 days to examine the fermentation process (Fig. 3). The simulated medium was used as a control group to observe the effects of the complex monosaccharides of SCG hydrolysate on microbial growth and production. SCG contains a variety of compounds beyond lignocellulose and according to the data, it also contains 6.7–13.6% proteins, 10–20% lipids, and minerals by weight (29). Consequently, when using SCG hydrolysate as a bacterial medium, no additional nutrients were added to fulfill the growth requirements of bacteria.

Figure S3 shows the changes in cell concentrations within 24 h for the different methods of cultivation. In the simulated medium, due to the absence of inhibitors, the lag phase between WT and EB showed no significant difference. The lack of inhibitors of bacterial growth enables the nutritional needs of the strain to be met by glucose metabolism, accounting for the absence of a significant difference in the lag phase between WT and EB. However, in the SCG hydrolysate, the presence of inhibitors increased the lag phases of both WT and EB. Nevertheless, irrespective of the method of inoculation, EB completed its lag phase by 12 h, whereas WT required 18 h. This may have been due to glucose inhibition of other sugars in WT through carbon catabolite repression (CCR) (50). In contrast, in EB, highly active PMI and MAK are constitutively expressed and are unaffected by CCR. Therefore, EB can utilize glucose and mannose simultaneously for rapid generation of energy and carbon skeletons in the presence of inhibitors, thereby shortening the lag phase. This indicates that EB has a faster growth rate in mannose-rich media. The results demonstrate that the introduction of MAK and PMI could effectively enhance bacterial adaptation to mannose-rich media.

Figure 3A shows that no significant pH differences were observed between WT and EB in the simulated medium. In SCG medium, the pH decrease for both WT and EB was more rapid in the cultures inoculated with BC pellicle compared to those inoculated with broth. The pH trend across all media was nearly identical, stabilizing at a final pH of about 2.7. Residual sugar levels in the media showed more significant variations (Fig. 3B). Sugar consumption by EB inoculated with BC pellicle in SCG medium was quicker and more pronounced, marking a significant divergence from other trends. At the end of fermentation, the sugar consumption rate was approximately 52% (Table 2), likely because EB does not have enhanced metabolic pathways for galactose and arabinose, as same as WT. Additionally, the method used for cultivation in this study involved static culture in flasks, in which the pH could not be adjusted conveniently by the addition of alkali solutions to optimize fermentation outcomes. *K. xylinus* is an acetic acid bacterium and produces large amounts of organic acids, such as acetic acid or gluconic acid, during growth (2), thereby reducing the pH of the culture medium (Fig. 3A) and inhibiting bacterial activity. Similar results have also been observed in other studies on biorefining. Chen et al. reported the production of BC from sweet sorghum bagasse, with a sugar consumption rate of approximately 60%, where the primary monosaccharides in the hydrolysate were found to be glucose and xylose, with xylose accounting for about 28% and glucose for about 68%. The rate of sugar conversion to crude BC (unpurified) was approximately 61%. After the collection of BC, it was found that glucose was not fully utilized (5). Future studies will further optimize both the strains and fermentation methods to enable full utilization of nutrients. This study focused on the metabolic enhancement of mannose; thus, other sugars' utilization was ignored. The relative conversion differences of mannose are shown in Table 2. EB-SCG(P) exhibited the highest mannose conversion rate (BC yield per gram of sugar × 100%), showing significant differences compared to other cultivation methods. The relative conversion rate of mannose can reflect the enhanced mannose metabolism of EB, but more accurate analysis of all saccharide metabolism will be conducted in the next study. The second-highest sugar consumption rate was seen in EB-SCG(L) (47%), with a mannose conversion rate of 28%, showing significant differences compared to all cultivation methods of WT. Although initially lacking the protective effects of BC for the cultures that inoculation of broth in simulated and SCG media, EB still demonstrated superior carbon source utilization in SCG medium compared to WT. The introduction of foreign genes evidently improved the mannose utilization capability of the bacteria. WT-simulated, which had the lowest sugar consumption rate (28%) and conversion rate (15%), with a mannose conversion rate of approximately 8%, which aligns with expectations. This phenomenon likely suffered from a weak mannose metabolic pathway, with only 4.12 g/L glucose in the medium and no growth-promoting compounds like caffeine. The BC cumulative yield is a key focus of this study. After 15 days of fermentation, EB-SCG(P) achieved the highest yield at 3.80 g/L (Fig. 3), significantly higher than the control group (*P* < 0.05). EB was observed to thrive better in a mannose-rich environment, whether in semi-synthetic or SCG medium. The comparison between simulated and SCG media also indicates that SCG medium can promote the conversion of sugar to BC (Table 2), further demonstrating the superiority of the SCG medium.

**TABLE 2:** Sugar consumption rate and conversion rate and crystallinity of BC^*a*^

Type	Sugar consumption rate (%)	Conversion rate (g BC/g sugar)	Mannose conversion rate (g BC/g mannose)	Crystallinity of BC (%)
WT-simulated medium	28	0.15 ± 0.02	0.08 ± 0.02	50.81 ± 7.64
EB-simulated medium	37	0.18 ± 0.06	0.12 ± 0.04	55.88 ± 5.17
WT-SCG (L)	28	0.30 ± 0.01	0.16 ± 0.02	60.72 ± 7.05
EB-SCG (L)	47	0.33 ± 0.02	0.28 ± 0.03	63.51 ± 3.19
WT-SCG (P)	31	0.37 ± 0.04	0.22 ± 0.05	63.10 ± 4.15
EB-SCG (P)	52	0.37 ± 0.02	0.34 ± 0.01	67.83 ± 6.28

^*a*^ WT-simulated medium, wild-type bacteria fermented in simulated medium. EB-simulated medium, engineered bacteria fermented in simulated medium. WT-SCG (L), wild-type bacteria were inoculated in the form of liquid and fermented in SCG hydrolysate medium. EB-SCG (L), engineered bacteria were inoculated in the form of liquid and fermented in SCG hydrolysate medium. WT-SCG (P), wild-type bacteria were inoculated in the form of BC pellicle and fermented in SCG hydrolysate medium. EB-SCG (P), engineered bacteria were inoculated in the form of BC pellicle and fermented in SCG hydrolysate medium. Sugar consumption rate = [total sugar consumption concentration (g/L)/total initial sugar concentration (g/L)] × 100%. Conversion rate = BC cumulative yield (g/L)/total sugar consumption concentration (g/L). Mannose conversion rate = BC cumulative yield (g/L)/initial mannose concentration (g/L).

The yields of BC listed in Table S1 are not entirely comparable since different purification processes and operating procedures, as well as various drying methods, including different drying temperatures, were used. In addition, yield is influenced by various factors, including the strain, reactor, and fermentation process. The industrialization of biomass biorefinement requires the implementation of multiple measures. This study did not seek high-yield strains nor optimization of the fermentation process but instead merely compared control and experimental groups using SCG as a mannose-rich feedstock model, demonstrating the improved efficiency of mannose conversion by the engineered bacteria and thereby the feasibility of enhancing BC production by reinforcing the metabolism of the carbon source, representing a novel strategy/concept. This study focused primarily on the construction of engineered strains with enhanced ability to metabolize mannose, aiming to supplement research on biomass media for BC production from the perspective of synthetic biology. Subsequent studies will further optimize the strain and fermentation process to facilitate industrial application.

Furthermore, we discovered that inoculating with a BC pellicle was an effective strategy to mitigate the impact of inhibitors. Beyond traditional detoxification methods aimed at reducing inhibitor concentrations as much as possible, leveraging the inherent protective qualities of BC itself offers another potential solution. During the late fermentation stage, free cells remain unchanged significantly (Fig. 3E), which is attributed to the limited oxygen supply in the culture medium during static cultivation. Most bacteria adhere to the BC pellicle for survival, with a smaller number of free-floating bacteria (Fig. 3). This finding is consistent with previous literature reports that BC forms at the air-liquid interface of the culture medium and gradually thickens over time (51). In the hydrolysate-based medium, there is a significant difference in the number of cells of EB that were inoculated in different forms (Fig. 3F), which corresponds to the yield of BC. In SCG media, the number of cells in BC significantly increased (*P* < 0.05) from day 7 to day 11, indicating that BC is produced in large quantities during this logarithmic growth phase. After day 11, the cell growth rate gradually slows down, entering the stationary phase. Observing changes in cell numbers can indirectly assess BC yield at different fermentation stages, even post-fermentation, which holds significant importance for industrial BC production. At the end of fermentation, the number of free cells in the hydrolysate media was lower (Fig. 3E), while the number of cells within the BC pellicle was higher (Fig. 3F). Pellicle inoculation yielded a significantly higher (*P* < 0.05) cell count in the produced BC compared to liquid inoculation (Fig. 3F). These phenomena demonstrate that BC could protect cells and mitigate negative impacts when inhibitors are present. Numerous studies have also reported similar conclusions related to BC protection. For instance, the cellulose produced by acetic acid bacteria on the surface of decomposing fruit shields bacterial cells from UV radiation, enhancing growth and metabolism. The acid resistance of acetic acid bacteria is also enhanced by the protective effect of BC (52). Yoo et al. found that cellulose offers bacterial protection against osmotic and chlorine stress (53). In the context of SCG hydrolysate, the BC matrix of pellicle provides a buffering effect against inhibitors and safeguards bacterial growth in the early stages of fermentation.

In the present study, the concept of strain-medium adaptability was put forward. Mannose, as a crucial constituent of hemicellulose, is extensively distributed in biomass resources. An exploration of the synthesis of BC using seven types of sugars as carbon sources has verified that *K. xylinus* exhibited the highest utilization capacity for glucose, while its utilization of mannose was less than 30% in comparison to that of glucose (2). In the case of media containing a mixture of multiple sugars, the researchers observed that although the consumption of other sugars increased notably, the consumption of mannose in the multiple-sugar medium remained at the same level as that in the single-mannose medium, below 40% (2). The inefficient utilization of mannose presents a significant challenge when biomass resources are employed in large quantities as low-cost culture media. Although the issue of enhancing mannose metabolism has been investigated in other strains, there have been no reports regarding *K. xylinus*. The significance of this study lies in the construction of a mannose-enhanced *K. xylinus* strain and its application to an actual SCG hydrolysate for concept verification. Compared with the wild-type strain in the control group, the engineered strain demonstrated a significantly improved ability to utilize mannose.

### Characterization of BC

**Fig 4:**
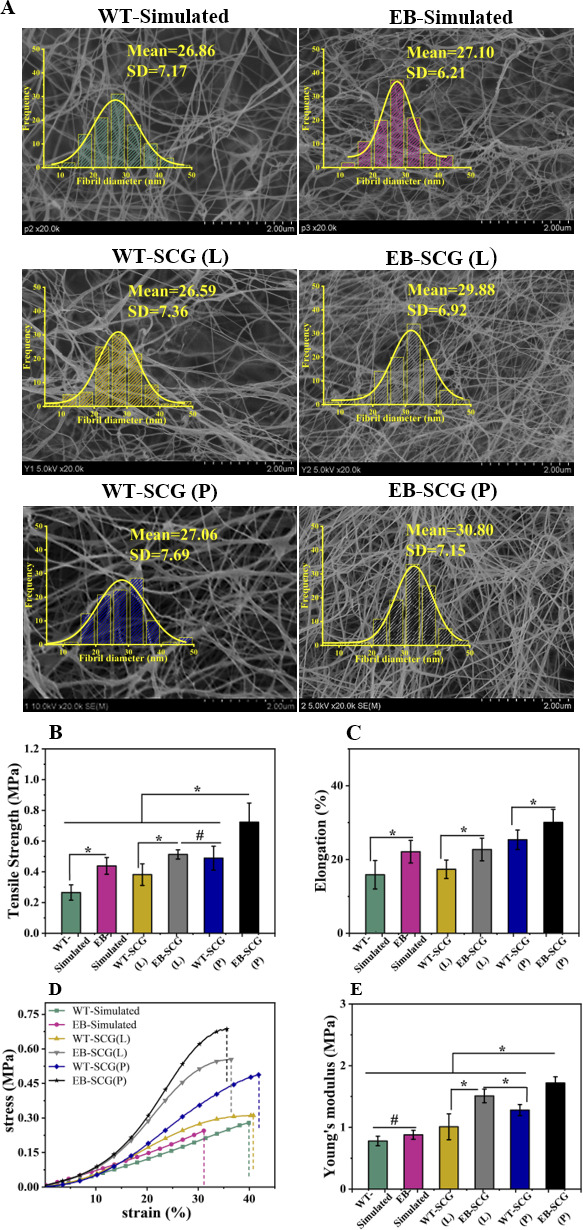
(**A**) SEM micromorphology (20,000× magnification) and fiber diameter of BC produced in simulated medium and SCG enzymatic hydrolysate medium with inoculation in the form of broth and BC pellicle of wild-type strain and engineered strains, respectively. Mechanical properties of BC produced by wild-type strain and engineered strain in simulated and SCG enzymatic hydrolysate medium, respectively. (**B**) Tensile strength, (**C**) elongation, (**D**) stress–strain curves, and (**E**) Young’s modulus. L, liquid inoculation. P, BC pellicle inoculation. Simulated, simulated medium. *P < 0.05; #*P* > 0.05.

The BC produced by both WT and EB strains in various culture media looked similar, typically forming a complete white pellicle on the surface of the culture medium. Scanning electron microscope images depicted in Fig. 4A were used to measure the fiber diameter. All BC samples displayed a three-dimensional network structure made up of nanoscale fibers. The fibers produced by EB-SCG(P) had the largest diameter (30.80 nm) and were significantly thicker (*P* < 0.05) than those of the other samples. The fiber diameters of the other four samples did not show significant variations. Under the same culture environment, the fiber diameter of EB is larger than that of WT, and the robust fiber also proves the adaptability of EB in SCG medium to a certain extent.

Figure S4 illustrates the FT-IR spectra of the BC. Characteristic peak sites 1,158, 2,900, and 3,550 cm^−1^ were observed. The peak around 1,158 cm^−1^ is associated with the glycosidic linkages in cellulose. The peaks at approximately 3,350 and 2,900 cm^−1^ are indicative of the OH^−^ groups in cellulose and the C-H stretching vibrations in cellulose, respectively (2). The results were consistent across all samples and aligned with the characteristics of BC. Throughout BC production, any compounds from the SCG medium in the pellicle can be removed during the purification process, ensuring that the BC produced is suitable for use in various applications without any contamination.

Table 2 displays the XRD results for BC derived from all culture methods. The data indicate that the crystallinity of BC produced by the EB strain is consistently higher than that produced by the WT strain across all conditions, with notable differences between them. Specifically, EB-SCG(P) exhibited the highest crystallinity (67.83%), followed by EB-SCG(L) (63.51%). Furthermore, the performance of BC produced in SCG medium is significantly greater than that produced in simulated medium, indicating that the components in the SCG medium, such as caffeine, would have a positive effect on the production and performance of BC.

The mechanical properties of BC are illustrated in Fig. 4. The data revealed that the tensile strength of BC produced by the EB strain in simulated medium was substantially higher than that produced by the WT strain. In the SCG hydrolysate, the tensile strength of EB-SCG(L) is significantly higher than that of WT-SCG(L) (*P* < 0.05). The tensile strength of EB-SCG(P) was the highest, being 1.4 times greater than WT-SCG(P), 1.4 times higher than that from liquid inoculation, and 1.7–2.1 times greater than that produced in simulated medium (Fig. 4B). A similar pattern was observed in the elongation analysis. The elongation value of EB-SCG(L) was 31% higher than that of WT-SCG(L), and the elongation value of EB-SCG(P) was 19% higher than that of WT-SCG(P). In the same culture conditions, the elongation of BC produced by EB was greater, indicating that the material could undergo larger deformations under stress (Fig. 4C). The stress-strain curves and Young’s modulus for six BC samples are displayed in Fig. 4D and E. WT-SCG(P) exhibited the steepest stress-strain curve and the highest Young’s modulus, significantly differing from the other BC samples (*P* < 0.05). Clearly, the BC produced by WT-SCG(P) demonstrated the best mechanical properties, capable of withstanding greater forces. Conversely, the Young’s modulus for the two BC samples produced in simulated medium was the lowest among all samples, showing significant differences.

The current findings indicate that BC produced through pellicle inoculation exhibits superior performance, particularly when produced in SCG medium, which displays the strongest mechanical properties. BC produced by the engineered bacterium *K. xylinus* CGMCC 31806, which harbors the *pmi-mak* gene, shows excellent compatibility with SCG medium.

Although the development of culture media and genetic modification of strains are commonly used in BC production to increase yield and reduce costs, research combining both approaches is rare. Currently, there is only one known case that enhances lactose metabolism pathways to produce BC by using whey (10). Whey was considered a byproduct of dairy production, rich in essential amino acids and bioactive peptides. Now, whey protein is widely used in food and beverages, such as milk powder and sports drinks, and its high-value utilization has become well-established (54). Therefore, whey should no longer be regarded as low-value biomass, and developing it as a culture medium is no longer meaningful. It is worth noting that most biomass resources in nature exist in the form of cellulose, hemicellulose, or lignocellulose. Lignocellulose hydrolysates contain various types of monosaccharides. However, the common BC-producing strain, *K. xylinus*, is proficient at utilizing glucose or fructose and has weak capabilities for other sugars. This leads to low utilization of nutrients in biomass media, resulting in resource waste and increased costs. In further studies, metabolic pathways for other sugars such as xylose and arabinose, which are rich in lignocellulose, will also be introduced into *K. xylinus*. This may enable the strain to fully utilize biomass media, thereby could make BC production cleaner.

Biomass is diverse in species, with different biorefining pathways possessing unique technical characteristics and applications. For example, the biofuel pathway produces fuels such as ethanol from waste biomass, posing no threat to food security or forest resources, thereby achieving a win-win situation for food and the environment (55). BC is a typical high-value-added bio-based material with broad application prospects. Developing SCG for BC production would promote the development of the circular economy, achieving maximum utilization of resources.

In this study, we focused on mannose, an important component of hemicellulose, and constructed a production strain with enhanced ability to metabolize mannose. Using SCG as an example, the findings demonstrated the potential of the engineered strain in applications involving production from natural biomass. Despite the production of BC and the efficiency of SCG hydrolysis being relatively lower than those reported in other studies, further improvements can be achieved through further optimization of the strain and fermentation process in the future. The successful application of strain *K. xylinus* CGMCC 31806 in the spent coffee ground medium verified the feasibility of the concept of “strain-medium matching.” This implies that the utilization of other mannose-rich biomass resources, such as soft wood and konjac, for BC production may further increase yields and reduce environmental pollution by fermentation effluents. With the full utilization of plentiful low-cost biomass and increases in BC production, the production of BC may become more economically viable. We will continue to develop the ability of *K. xylinus* to utilize other types of sugar, reduce the limitations of raw materials in culture media, and promote the green and low-cost production of BC.

### Conclusion

This study pioneers the integration of the mannan component of SCG with the metabolic enhancement of engineered bacteria, surpassing the constraints of single cost control strategies through the collaborative optimization of “Feedstock-Strain-Process.” The *mak-pmi* fusion protein gene of *E. coli* was introduced into *K. xylinus*, constructing a new strain, *K. xylinus* CGMCC 31806, which is better adapted to a mannose-culture environment. The findings revealed that the yield and BC properties of the engineered bacterium grown in SCG medium significantly outperformed those of the parent strain. Additionally, the BC protection mechanism offers a novel approach for lignocellulosic hydrolysate fermentation. Improved BC mechanical properties expand its functional material applications, offering fresh insights for industrial BC production. SCG serves as a raw material model, laying the foundation for the utilization of other mannose-rich biomass. This approach may reduce raw-material carbon emissions, align with circular economy goals, and foster economic-environmental synergy in the BC industry.

## SUPPLEMENTAL MATERIAL

Supplemental materials aem.02439-25-s0001.docx Supplemental materials and methods, Tables S1 to S3, and Fig. S1 to S4.

ASM does not own the copyrights to Supplemental Material that may be linked to, or accessed through, an article. The authors have granted ASM a non-exclusive, world-wide license to publish the Supplemental Material files. Please contact the corresponding author directly for reuse.
