# Supplementary material for: Enhanced bacterial cellulose production from spent coffee grounds: a dual-strategy synergy via mannose metabolism and pellicle inoculation
Source: Appl Environ Microbiol. 2026 May 5;92(6):e02439-25. doi: 10.1128/aem.02439-25 (PMC13274416; doi:10.1128/aem.02439-25)
Supplement: Supplemental materials — and methods, Tables S1 to S3, and Fig. S1 to S4. [file aem.02439-25-s0001.docx]

**Supplementary Information**

**Enhanced Bacterial Cellulose Production from Spent Coffee Grounds: A Dual-Strategy Synergy via Mannose Metabolism and Pellicle Inoculation**

1. **Materials and methods**

**1.1 Bacterial strains, plasmids, and growth conditions**

The *K. xylinus* 23770 used in this study was from American Type Culture Collection (Manassas, VA, USA). Plasmid (pBBR1MCS-2) was sourced from laboratory storage. Bacteria were cultured in Hestrin & Schramm medium (1), containing 40 g/L glucose, 10 g/L peptone, 3 g/L yeast extract, 6.75 g/L Na_2_HPO_4_·12H_2_O and citric acid, with an initial pH of 5.2. In mannose-based medium, mannose replaced glucose while other components remained unchanged. Kanamycin was added to the culture medium of the engineered strain at 50 mg L^−1^. Unless specified otherwise, the experiments were performed using static culture at 30^o^C. All reagents for the medium were supplied from Sinopharm Chemical Reagent Co. (Shanghai, China).

**1.2 Variations in cell concentration of strains within 24 h**

Strains EB and WT were inoculated into simulated medium and hydrolysate according to the method mentioned in section 2.6, with samples taken every 3 h. The cell count of the samples was analyzed using the fluorescence staining method established by Zou et al. (2).

**1.3 X-ray diffractometer (XRD) analysis**

The purified BC samples were freeze-dried for XRD analysis (D/max-2550VB+/PC, Rigaku, Tokyo, Japan).

**Table S1. Cellulosic and lignocellulosic wastes used for BC production**

| **Source** | **BC productivity**  **(g·L^-1^·d^-1^)** | **BC processing method** | **Strain** | **Reference** |
| --- | --- | --- | --- | --- |
| Spent coffee grounds | 0.253 | 1% NaOH solution at 80 ^o^C for 2 d, wash with DI water until pH is neutral. Dry the membrane at 105 ^o^C until weight is constant. | Recombinant *Komagataeibacter xylinus* CGMCC 31806 | This study |
| Waste dyed cotton fabrics hydrolysate | 1.28  (Not purified*) | DI water cleaning, dried at 105 ^o^C | *Gluconacetobacter xylinus* | (3) |
| Waste cotton textiles | 0.134 | Immerse in 1N NaOH solution at 95 ^o^C for 20 min. DI water cleaning, dried at 70 ^o^C | *Gluconacetobacter xylinus* | (4) |
| Waste fiber sludge sulfate and sulfite fiber sludges | 0.786  (Not purified*) | Filtration, dried at 105 ^o^C | *Gluconacetobacter xylinus* ATCC 23770 | (5) |
| Corn stalk | 0.409 | Immerse in 0.5 mol/L NaOH solution at 80 ^o^C for 90 min. DI water cleaning, dried at 80 ^o^C | *Acetobacter xylinum* ATCC 23767 | (6) |
| Wheat straw | 1.23  (Not purified*) | G_3_ crucible filtration, dried at 105 ^o^C | *Gluconacetobacter xylinus* ATCC 23770 | (7) |
| Sugarcane straw | 0.301 | Immerse in 0.1 mol/L NaOH solution at 80 ^o^C for 90 min. DI water cleaning, dried at 80 ^o^C | *Komagataeibacter xylinus* ATCC 11142 | (8) |
| Sweet sorghum | Stalk 0.303  Leaf 0.423  Root 0.380  Juice 0.145 | Immerse in 0.5 mol/L NaOH solution at 80 ^o^C for 1h. DI water cleaning, dried at 80 ^o^C | *Acetobacter xylinum* ATCC 23767 | (9) |
| Sweet sorghum bagasse | 0.710  (Not purified*) | DI water cleaning, dried at 105 ^o^C | *Komagataeibacter xylinus* ATCC 23770 | (10) |
| Cashew tree residues | 0.857 | Immerse in 0.1 M NaOH solution at 80 ^o^C for 45 min. DI water cleaning, dried at 38 ^o^C | *Komagataeibacter rhaeticus* | (11) |
| Red maple | 0.005 | 1% NaOH boiling for 30 min, followed by DI water rinse, and freeze-drying. | *Acetobacter xylinus*  23769 | (12) |
| Pecan nutshell | 0.101 | 0.5M NaOH boiling for 30 min, followed by DI water rinse, and freeze-drying. | *Gluconacetobacter entanii* | (13) |
| *Caragana Korshinskii* Kom | 0.657 | 0.5M NaOH treatment, followed by DI water rinse, and freeze-drying. | *Gluconacetobacter xylinus* CGMCC 2955 | (14) |
| Oat hulls | 0.029-0.917 | 0.5M NaOH treatment, followed by DI water rinse, and freeze-drying. | *Medusomyces gisevii* Sa-12 | (15) |
| Elephant grass | 0.457 | 1M NaOH boiling for 30 min, followed by DI water rinse, and freeze-drying. | *Gluconacetobacter xylinus* CH001 | (16) |
| Coffee cherry husk | 0.586 | Immerse at room temperature in a 1N NaOH solution for 1 d, wash with DI water until neutral. Dry the membrane on filter paper at 60 ^o^C until the weight is constant. | *Gluconacetobacter*  *hansenii* UAC09 | (17) |
| Mulberry leaves | 0.291  (Not purified*) | DI water immersion for 3 days, followed by freeze-drying. | *Acetobacter xylinum* NUST 4.2 | (18) |
| Spruce hydrolysate | 0.586  (Not purified*) | DI water cleaning, dried at 105 ^o^C | *Gluconacetobacter xylinus* ATCC 23770 | (19) |
| Dry olive mill residue | 0.213 | Immerse in 0.5 M NaOH solution at 90 ^o^C for 30 min. DI water cleaning, dried at 105 ^o^C | *Gluconacetobacter sacchari* | (20) |
| Industrial hardwood | 0.068 | Immerse in 0.5 M NaOH solution at 90 ^o^C for 30 min. DI water cleaning, dried at 105 ^o^C | *Gluconacetobacter sacchari* | (21) |
| Acerola byproduct | 0.267 | Immerse in 0.1 M NaOH solution at 50 ^o^C for 24 h. DI water cleaning, dried at 105 ^o^C | *Komagataeibacter rhaeticus* | (22) |
| Rice husk | Aerated ~0.09  Static ~0.13 | Centrifuge (5000 rpm for 15 min), Immerse in 0.1 M NaOH solution for 24 h. Wash with DI water, freeze-dry. | *Acetobacter xylinum*  ATCC 23769 | (23) |

*‘Not purified’ means BC was not treated with NaOH, but only immersed or rinsed with DI water.

**#Table S2. Current utilization status of SCG**

| **Hydrolysis method /Pretreatment** | **Enzyme** | **Sugar recovery rate^*^** | **Product** | **Reference** |
| --- | --- | --- | --- | --- |
| Acid hydrolysis：  7.4% H_2_SO_4_ (w/v), 1:10^#^ (0.74 g 100% H_2_SO/g SCG, 95 ^o^C, 120 min. | - | Sugar yield：31% | Reducing sugar | (24) |
| Acid hydrolysis：  1.0% H_2_SO_4_ (w/v), 1:10^#^ (0.1 g 100% H_2_SO/g SCG), 163 ^o^C, 45 min | - | Efficiency:  100% galactan 77.4% mannan 89.5% arabinan | Water-soluble polysaccharides | (25) |
| Acid hydrolysis:  5.5% H_2_SO_4_ (w/v), 1:5^#^ (0.3g 100% H_2_SO/g SCG), 100 ^o^C for 2h | Simultaneous saccharification and fermentation:  15% solid loading, 50 ^o^C, 125 rpm, 72 h, enzyme ACCELLERASE^®^ 1500 (0.02 mL/g SCG) +*Lipomyces starkeyi* | 81.5% | Microbial oil | (26) |
| Microwave water extraction:  1:10^#^, 200 ^o^C was attained in 3 min using microwave and maintain for 2 min | - | 42% | Water-soluble polysaccharides | (27) |
| Sequential microwave superheated water extraction:  1:30^#^, 200 ^o^C in 3 min and maintained 2 min for 4 cycles. The fifth cycle reached 230 ^o^C in 3 min and maintained 2 min. | - | 3rd: 48% recovery  4th: 56% recovery  5th: 69% recovery | Mannan | (28) |
| Ethanol pretreatment:  1:5^#^, mixture was heated at 150 ^o^C for 2 h | Cellulase (17.7 mg/g SCG)  Pectinase (16.0 mg/g SCG) | 95.3% | Mannose and bioethanol | (29) |
| Acid-chlorite pretreatment:  A 1:2 mixture of alcohol and benzene was used to extract SCG. 1:10^#^, SCG was incubated with 4 g NaClO_2_and 0.8 mL HAc at 80 ^o^C for 1h | Celluclast 1.5 L (8.4mg/g SCG), Pectinex SP-L (8.4mg/g SCG) Xylanase (4.8 mg/g SCG), 45 ^o^C, 24h | 78% | Bio-sugar and bioethanol | (30) |
| Atmospheric air plasma and FeCl_3_ Pretreatment:  SCG was treated for 2 min at a voltage of 70 kV in an environment of 1% H_2_SO_4_ (w/v), 1:20^#^ | Cellulase (23.1 FPU/g SCG), Hemicellulase (5.33 U/g SCG), pH 6.7, 50 ^o^C, 48h | 49.6% | Ethanol | (31) |
| Diluted acid Pretreatment:  1:6^#^, SCG was treated at 121°C for 30 min in a 1.76% H₂SO₄ (w/v) solution | Accellerase 1500^®^ (0.3 mL/g SCG), pH 5.0, 50 ^o^C, 24h | 72.8%-89.1% | Lactic acid | (32) |
| Popping Pretreatment:  1:5^#^, SCG underwent a poping pretreatment for 10 min at 1.47 MPa | Celluclast 1.5 L  (18.3 mg/g SCG), Pectinex SP-L  (11.6 mg/g SCG), pH 4.8, 37 ^o^C, 48h | 85.6% | Ethanol | (33) |
| Delignification and Defatting Pretreatment:  SCG was heated for 3 h at 80 ^o^C in a solution of 1% sodium chlorate and 1% acetic acid at 1:10^#^, then defatted with hexane for 6 h | Cellulase (4.1 mg/g SCG),Pectinase (4.1 mg/g SCG), pH 4.8, 45 ^o^C, 24h | 92.5% | Manno-oligosaccharides and mannose | (34) |
| Acid Pretreatment：  70% H_2_SO_4_ (w/v), 1:1^#^ (0.7 g 100% H_2_SO/g SCG), mixture at 30 ^o^C for 1h | Cellulase (2000 U/L), Hemicellulase (2000 U/L), pH 5.0, 50 ^o^C, 24h | 84.7% | Bacteria cellulose | This study |

# solid-to-liquid ratio

* Sugar recovery rate refers to the ratio of the sugar yield obtained through hydrolysis to the total sugar content of the SCG dry matter.

**Table S3. Strains, plasmids and primers used**

| Strain or Plasmid | Relevant genotype, mode of construction, or feature | Reference or source |
| --- | --- | --- |
| *E. coli* K-12 | Wild type | (35) |
| *K. xylinus* ATCC 23770 | Wild type | Laboratory stock |
| *K. xylinus* CGMCC 31806 | Introducing pBBR1MCS:*pmi*-*mak* | This study |
| *K. xylinus* PMI | Introducing pBBR1MCS:*pmi* | This study |
| *K. xylinus* MAK | Introducing pBBR1MCS:*mak* | This study |
|  |  |  |
| Plasmids |  |  |
| pBBR1MCS-2 | Kan; MCS; lacZα; Pnk | (36) |
| pBBR1MCS:*pmi* | pBBR1MCS-2 with *pmi* as *Xho*I-*Nde*I fragment | This study |
| pBBR1MCS:*mak* | pBBR1MCS-2 with *mak* as *NdeI*-*Bam*HI fragment | This study |
| pBBR1MCS:*pmi*-*mak* | pBBR1MCS-2 with *pmi* and *mak* as *Xho*I-*Bam*HI fragment | This study |
|  |  |  |
| Primers |  |  |
| F_Pnk:PMI_*Xho*I | CTCGAGCCGGAATTGCCAGCTGGGG |  |
| R_Pnk:PMI_*Nde*I | CATATGCAGCTTGTTGTACGCGCT |  |
| F_MAK_*NdeI* | GGTACCAAGGAAGGTCGACTCATGCG |  |
| R_MAK_*Bam*HI | GGATCCTTAATGATGATGATGATGG |  |
| F_Pnk:MAK_*Xho*I | CTCGAGCCGGAATTGCCAGCTGGGG |  |
| R_Pnk:MAK_*Bam*HI | GGATCCTTAATGATGATGATGATGG |  |

Kan, kanamycin resistance. Pnk, Pnk promoter.

In the primers, restriction sites are indicated by underlining.


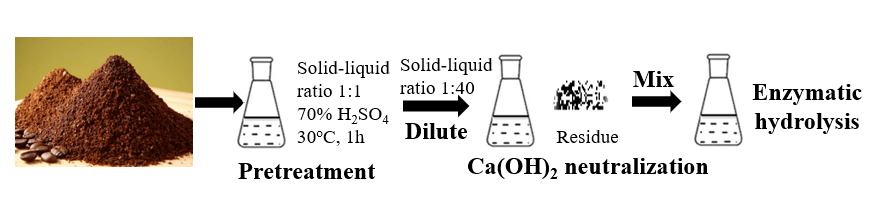


Fig. S1. Schematic diagram of enzymatic hydrolyses of SCG.


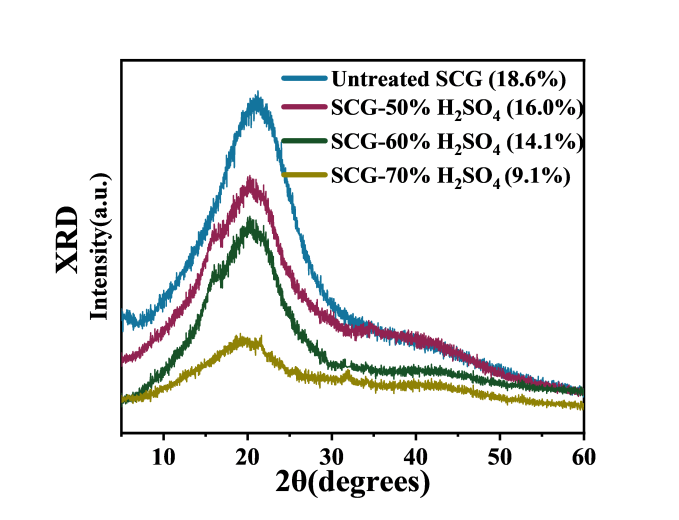


Fig. S2. XRD test curves of SCG treated with 50%, 60%, and 70% sulfuric acid. The crystallinity is denoted in the legend.

Fig. S3. Variations in the cell concentration of wild-type and engineered strains within 24 h in the simulated medium and the SCG hydrolysate medium. *p < 0.05; ** p < 0.01; # p > 0.05.


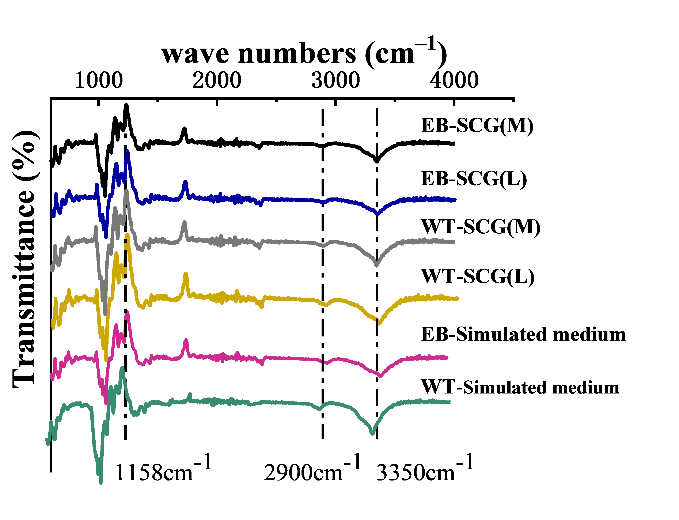


Fig. S4. FT-IR spectra of the BC synthesized by the wild-type strain and the engineered strain in the simulated medium and the SCG hydrolysate medium.

**References:**

1. Chen G, Wu G, Chen L, Wang W, Hong FF, Joensson LJ. 2019. Comparison of productivity and quality of bacterial nanocellulose synthesized using culture media based on seven sugars from biomass. *Microb Biotechnol* 12:677-687.

2. Zou X, Zhang S, Chen L, Hu J, Hong FF. 2020. Determination of live and dead Komagataeibacter xylinus cells and first attempt at precise control of inoculation in nanocellulose production. *Microb Biotechnol* 13:458-469.

3. Guo X, Chen L, Tang J, Joensson LJ, Hong FF. 2016. Production of bacterial nanocellulose and enzyme from [AMIM]Cl-pretreated waste cotton fabrics: effects of dyes on enzymatic saccharification and nanocellulose production. *J Chem Technol Biotechnol* 91:1413-1421.

4. Kuo C-H, Lin P-J, Lee C-K. 2010. Enzymatic saccharification of dissolution pretreated waste cellulosic fabrics for bacterial cellulose production by Gluconacetobacter xylinus. *J Chem Technol Biotechnol* 85:1346-1352.

5. Cavka A, Guo X, Tang S-J, Winestrand S, Joensson LJ, Hong F. 2013. Production of bacterial cellulose and enzyme from waste fiber sludge. *Biotechnol Biofuels* 6:25.

6. Cheng Z, Yang R, Liu X, Liu X, Chen H. 2017. Green synthesis of bacterial cellulose via acetic acid pre-hydrolysis liquor of agricultural corn stalk used as carbon source. *Bioresour Technol* 234:8-14.

7. Hong F, Zhu YX, Yang G, Yang XX. 2011. Wheat straw acid hydrolysate as a potential cost-effective feedstock for production of bacterial. *J Chem Technol Biotechnol* 86:675-680.

8. Dhar P, Pratto B, Goncalves Cruz AJ, Bankar S. 2019. Valorization of sugarcane straw to produce highly conductive bacterial cellulose / graphene nanocomposite films through in situ fermentation: Kinetic analysis and property evaluation. *J Cleaner Prod* 238:117859.

9. Wang Q, Nnanna PC, Shen F, Huang M, Tian D, Hu J, Zeng Y, Yang G, Deng S. 2021. Full utilization of sweet sorghum for bacterial cellulose production: A concept of material crop. *Ind Crops Prod* 162:113256.

10. Chen G, Chen L, Wang W, Hong FF. 2018. Evaluation of six ionic liquids and application in pretreatment of sweet sorghum bagasse for bacterial nanocellulose production. *J Chem Technol Biotechnol* 93:3452-3461.

11. Pacheco G, Nogueira CR, Meneguin AB, Trovatti E, Silva MCC, Machado RTA, Ribeiro SJL, da Silva Filho EC, Barud HdS. 2017. Development and characterization of bacterial cellulose produced by cashew tree residues as alternative carbon source. *Ind Crops Prod* 107:13-19.

12. Erbas Kiziltas E, Kiziltas A, Gardner DJ. 2015. Synthesis of bacterial cellulose using hot water extracted wood sugars. *Carbohydr Polym* 124:131-138.

13. Dorame-Miranda RF, Gamez-Meza N, Medina-Juarez LA, Ezquerra-Brauer JM, Ovando-Martinez M, Lizardi-Mendoza J. 2019. Bacterial cellulose production by Gluconacetobacter entanii using pecan nutshell as carbon source and its chemical functionalization. *Carbohydr Polym* 207:91-99.

14. Li W, Zhang S, Zhang T, Shen Y, Han L, Peng Z, Xie Z, Zhong C, Jia S. 2021. Bacterial cellulose production from ethylenediamine pretreated Caragana korshinskii Kom. *Ind Crops Prod* 164:113340.

15. Skiba EA, Budaeva VV, Ovchinnikova EV, Gladysheva EK, Kashcheyeva EI, Pavlov IN, Sakovich GV. 2020. A technology for pilot production of bacterial cellulose from oat hulls. *Chem Eng J (Amsterdam, Neth)* 383:123128.

16. Yang XY, Huang C, Guo HJ, Xiong L, Li YY, Zhang HR, Chen XD. 2013. Bioconversion of elephant grass (Pennisetum purpureum) acid hydrolysate to bacterial cellulose by Gluconacetobacter xylinus. *J Appl Microbiol* 115:995-1002.

17. Rani MU, Appaiah KAA. 2013. Production of bacterial cellulose by Gluconacetobacter hansenii UAC09 using coffee cherry husk. *J Food Sci Technol* 50:755-762.

18. Chen J, Chen C, Liang G, Xu X, Hao Q, Sun D. 2019. In situ preparation of bacterial cellulose with antimicrobial properties from bioconversion of mulberry leaves. *Carbohydr Polym* 220:170-175.

19. Guo X, Cavka A, Jonsson LJ, Hong F. 2013. Comparison of methods for detoxification of spruce hydrolysate for bacterial cellulose production. *Microb Cell Fact* 12:93/1.

20. Gomes FP, Silva NHCS, Trovatti E, Serafim LS, Duarte MF, Silvestre AJD, Neto CP, Freire CSR. 2013. Production of bacterial cellulose by Gluconacetobacter sacchari using dry olive mill residue. *Biomass Bioenergy* 55:205-211.

21. Carreira P, Mendes JAS, Trovatti E, Serafim LS, Freire CSR, Silvestre AJD, Neto CP. 2011. Utilization of residues from agro-forest industries in the production of high value bacterial cellulose. *Bioresour Technol* 102:7354-7360.

22. Leonarski E, Cesca K, Zanella E, Stambuk BU, de Oliveira D, Poletto P. 2021. Production of kombucha-like beverage and bacterial cellulose by acerola byproduct as raw material. *LWT--Food Sci Technol* 135:110075.

23. Goelzer FDE, Faria-Tischer PCS, Vitorino JC, Sierakowski M-R, Tischer CA. 2009. Production and characterization of nanospheres of bacterial cellulose from Acetobacter xylinum from processed rice bark. *Mater Sci Eng, C* 29:546-551.

24. Go AW, Conag AT, Cuizon DES. 2016. Recovery of Sugars and Lipids from Spent Coffee Grounds: A New Approach. *Waste Biomass Valorization* 7:1047-1053.

25. Mussatto SI, Carneiro LM, Silva JPA, Roberto IC, Teixeira JA. 2011. A study on chemical constituents and sugars extraction from spent coffee grounds. *Carbohydr Polym* 83:368-374.

26. Wang H-MD, Cheng Y-S, Huang C-H, Huang C-W. 2016. Optimization of High Solids Dilute Acid Hydrolysis of Spent Coffee Ground at Mild Temperature for Enzymatic Saccharification and Microbial Oil Fermentation. *Appl Biochem Biotechnol* 180:753-765.

27. Passos CP, Coimbra MA. 2013. Microwave superheated water extraction of polysaccharides from spent coffee grounds. *Carbohydr Polym* 94:626-633.

28. Passos CP, Moreira ASP, Domingues MRM, Evtuguin DV, Coimbra MA. 2014. Sequential microwave superheated water extraction of mannans from spent coffee grounds. *Carbohydr Polym* 103:333-338.

29. Nguyen QA, Cho E, Trinh LTP, Jeong J-s, Bae H-J. 2017. Development of an integrated process to produce D-mannose and bioethanol from coffee residue waste. *Bioresour Technol* 244:1039-1048.

30. Kim HM, Choi Y-S, Lee D-S, Kim Y-H, Bae H-J. 2017. Production of bio-sugar and bioethanol from coffee residue (CR) by acid-chlorite pretreatment. *Bioresour Technol* 236:194-201.

31. Ravindran R, Sarangapani C, Jaiswal S, Cullen PJ, Jaiswal AK. 2017. Ferric chloride assisted plasma pretreatment of lignocellulose. *Bioresour Technol* 243:327-334.

32. Pleissner D, Neu A-K, Mehlmann K, Schneider R, Puerta-Quintero GI, Venus J. 2016. Fermentative lactic acid production from coffee pulp hydrolysate using Bacillus coagulans at laboratory and pilot scales. *Bioresour Technol* 218:167-173.

33. Choi IS, Wi SG, Kim S-B, Bae H-J. 2012. Conversion of coffee residue waste into bioethanol with using popping pretreatment. *Bioresour Technol* 125:132-137.

34. Nguyen QA, Cho EJ, Lee D-S, Bae H-J. 2019. Development of an advanced integrative process to create valuable biosugars including manno-oligosaccharides and mannose from spent coffee grounds. *Bioresour Technol* 272:209-216.

35. Sichwart S, Hetzler S, Broeker D, Steinbuechel A. 2011. Extension of the substrate utilization range of Ralstonia eutropha strain H16 by metabolic engineering to include mannose and glucose. *Appl Environ Microbiol* 77:1325-1334.

36. Kovach ME, Elzer PH, Hill DS, Robertson GT, Farris MA, Roop RM, II, Peterson KM. 1995. Four new derivatives of the broad-host-range cloning vector pBBR1MCS, carrying different antibiotic-resistance cassettes. *Gene* 166:175-176.
